# Effect of Supplementation with Hydroethanolic Extract of *Campomanesia xanthocarpa* (Berg.) Leaves and Two Isolated Substances from the Extract on Metabolic Parameters of Mice Fed a High-Fat Diet

**DOI:** 10.3390/molecules25112693

**Published:** 2020-06-10

**Authors:** Carla Maiara Lopes Cardozo, Aline Carla Inada, Claudia Andrea Lima Cardoso, Wander Fernando de Oliveira Filiú, Bernardo Barcelar de Farias, Flávio Macedo Alves, Mariana Bento Tatara, Júlio Henrique Rosa Croda, Rita de Cássia Avellaneda Guimarães, Priscila Aiko Hiane, Karine de Cássia Freitas

**Affiliations:** 1 Post Graduate Program in Health and Development in the Central-West Region of Brazil, Federal University of Mato Grosso do Sul-UFMS, Campo Grande 79070-900, MS, Brazil; carlinhalopescardozo@hotmail.com (C.M.L.C.); inada.aline@gmail.com (A.C.I.); rita.guimaraes@ufms.br (R.d.C.A.G.); priscila.hiane@ufms.br (P.A.H.); 2 Departament of Chemistry, State University of Mato Grosso do Sul, Dourados 79804-970, MS, Brazil; claudia@uems.br; 3 Pharmaceutical Science, Food and Nutrition Faculty, Federal University of Mato Grosso do Sul-UFMS, Campo Grande 79079-900, MS, Brazil; wander.filiu@gmail.com; 4 Diagnostic Medicine Laboratory-Scapulatempo, Campo Grande 79002-17, MS, Brazil; bacelarfaria@gmail.com; 5 Institute of Biosciences, Federal University of Mato Grosso do Sul-UFMS, Campo Grande 79079-900, MS, Brazil; flaurace@yahoo.com.br; 6 Health Science Research Laboratory, Federal University of Grande Dourados, Dourados 79804-970, MS, Brazil; marianabtatara@gmail.com (M.B.T.); juliocroda@gmail.com (J.H.R.C.); 7 School of Medicine Federal University of Mato Grosso do Sul, Campo Grande, Brazil Oswaldo Cruz Foundation, Campo Grande 79079-900, Brazil

**Keywords:** Brazilian plant, obesity, chalcones, flavanones, synergistic effect

## Abstract

There are still controversies regarding the correlation between the beneficial effects for health and the administration of isolated compounds or crude extracts in therapeutic applications. *Campomanesia xanthocarpa*, found in the Brazilian Cerrado, demonstrated beneficial effects in metabolic disorders associated with obesity. We investigated the effects of *Campomanesia xanthocarpa* hydroethanolic extract and two isolated substances from the extract (S1 and S2) in a diet-induced obesity (DIO) model. Male Swiss mice were divided into five groups: (1) American Institute of Nutrition (AIN-93M) diet, (2) high-fat diet (HF), (3) HF supplemented with *C. xanthocarpa* hydroethanolic leaf extract at 100 mg/kg (HFE), (4) HF supplemented with S1 at 1 mg/kg (HFS1) and (5) HF supplemented with S2 at 1 mg/kg (HFS2). The HFS1, HFS2 and HFE groups did not present decreasing body weight or visceral adiposity gain. No differences in glycemic and lipid parameters, or in the expression of protein content in two cytokines, interleukin-6 (IL-6) and anti-inflammatory (IL-10), were observed. Only the HFS1 group displayed decreased food intake. Even though substantial effects such as an improvement in obesity features or the metabolic and histological parameters promoted by S1, S2 and the extract were not observed, further investigations are necessary to evaluate the principal genes and protein expressions involved in regulating food behavior promoted by S1.

## 1. Introduction

Obesity is defined as excessive fat accumulation that may impair health [1] and is considered a risk factor for the development of noncommunicable diseases (NCDs), including cardiovascular diseases (CVDs) and diabetes [2]. According to the World Health Organization (WHO), about 41 million deaths have occurred due to NCDs, equivalent to 71% of all deaths in the world and 80% of all premature deaths [2], affecting individuals in low- and middle-income nations the most [2,3]. They are known as chronic diseases due to their long duration and the fact that they result from a set of genetic, physiological, environmental, and behavioral factors [2].

Central obesity, also known as abdominal obesity, is a crucial factor in the development of metabolic disturbances such as glucose intolerance, insulin resistance, hypertension, dyslipidemia, and nonalcoholic fatty liver disease (NAFLD), and all of these conditions are features of principal metabolic syndrome (MetS) [4]. Physical inactivity, age, genetic profile, obesity, and an unhealthy diet may be considered primary causes of MetS [5,6]. The evidence indicates that lifestyle modification is the first line of choice, and drug therapy is an important strategy for the management of MetS [7]. Current data, as reported by the World Obesity Federation (WOF), demonstrate that 1.2 trillion dollars will be spent on the treatment of obesity-associated diseases in 2025 [8], with synthetic drugs as the first choice for treatment. Several pharmaceutical products available on the market are used to treat obesity and its metabolic disturbances; however, some of these products have been associated with severe side effects [9]. For this reason, novel natural compounds with medicinal properties have been studied in order to develop effective agents that may be less toxic and produce fewer undesirable effects [10]. Thus, the acceptance of medicinal plants as an alternative therapy has grown considerably [11].

*Campomanesia xanthocarpa* (*C. xanthocarpa*), which belongs to the Myrtaceae family, is found in the Brazilian Cerrado and is used in Brazilian popular medicine for its antiulcerogenic [12], anti-inflammatory [10], antidiarrheal, and antimicrobial activity [13], and antioxidant potential [14]. A previous study by our group demonstrated that some *C. xanthocarpa* extracts were able to influence the metabolic dysfunctions commonly associated with obesity conditions [15]; in the present study, we observed that few studies so far have evaluated the effects of *C. xanthocarpa* extracts, such as by using leaf aqueous extracts [16,17] in animal models fed with diets that promote metabolic disturbances.

The difficulty in assessing which of the two therapeutic approaches—the use of extracts acting with a synergistic effect or isolated substances—provide the most beneficial effects on metabolic disturbances remains one of the challenges when using natural products to prevent or treat obesity. Bioactive compounds found in a variety of plants have attracted attention due to their pharmacological properties, and among these important substances are chalcones and flavanones, which belong to the flavonoid family [18]. Current studies have demonstrated that chalcones and flavanones exhibited metabolic effects in diet-induced obesity (DIO) animal models, such as reducing body weight gain and fat deposition in white adipose tissue, decreased plasma cholesterol, glucose, insulin and triglycerides levels, alleviated glucose tolerance and chronic inflammation in white adipose tissue and reduced fat content in the liver [19,20,21,22]. These metabolic effects promoted by chalcones and flavanones were also observed in in vitro cells, such as adipocytes, suppressing intracellular lipid accumulation and inhibiting adipocyte differentiation [23], and hepatocytes, ameliorating hepatic steatosis [24].

The present study is the first to evaluate the effects of *C. xanthocarpa* hydroethanolic leaf extract per se, and two isolated compounds, substance 1 (S1) (comprised of 2′,4′-dihydroxy-3′,5′-dimethyl-6′-methoxychalcone (chalcone)) and substance 2 (S2) (comprised of 5-hydroxy-7-methoxy-8-metylflavanone (flavanone)), were studied. The aim of this study is to observe the synergistic effect promoted by *C. xanthocarpa* hydroethanolic leaf extract (Ext) and the two substances (S1 and S2) isolated from the extract on obesity features and metabolic and histological parameters in high-fat diet (HFD)-fed mice. 

## 2. Results

### 2.1. S1, S2 and Ext Have Not Influenced on Decreasing Body Weight and Visceral Adiposity

Differences in body weight gain were not observed at the end of the first month of supplementation (data not shown). At the end of two months, the high-fat diet (HF) group presented a higher body weight gain compared to the American Institute of Nutrition (AIN-93M) group (data not shown). The HF group and the group fed HF supplemented with *C. xanthocarpa* hydroethanolic leaf extract (HFE) had an increased body weight gain at the end of three months of the experiment, while the groups fed HF supplemented with S1 (HFS1) and S2 (HFS2) did not present differences compared to the AIN-93M group. With regard to adiposity index and total visceral adipose tissue weight, the HF, HFE, and HFS2 groups obtained higher values; however, the HFS1 group did not show differences compared to the AIN-93M group. These findings suggest that the HFS1, HFS2 and HFE groups did not mitigate body weight and visceral fat gain; even though there were no differences among the supplemented groups in comparison to the HF group, it is important to emphasize that the HFE group displayed higher average values of final body weight, body weight gain, total visceral fat and adiposity index (Table 1).

### 2.2. S1 Attenuated Food Intake; However, S2 and Ext Did Not Decrease Food and Calorie Intake

The AIN-93M group presented a higher food intake compared to all groups at the end of the first (fourth week), second (eighth week), and third (13th week) month of the experiment. However, at the end of the experiment (13th week), the HFS1 group demonstrated lower food intake compared to the HF and AIN-93M groups. 

Even though the AIN-93M group had a higher food intake, the HF and HFE groups displayed an increased daily caloric intake and weight gain per caloric intake coefficient (WGPCI). However, the HFE group obtained a higher feed efficiency index (FEI) compared to the AIN-93M group, demonstrating that the crude extract did not have an influence on food behavior and calorie intake. Lower average values of FEI and WGPCI values were observed in the HFS1 and HFS2 groups. Taken together, these data suggest that the supplemented groups do not displayed differences in calorie intake, FEI and WGPCI; however, the HFS1 group displayed a decreased food intake at the end of supplementation, while HFS2 and HFE were not able to present this effect (Table 2).

**Figure 1:**
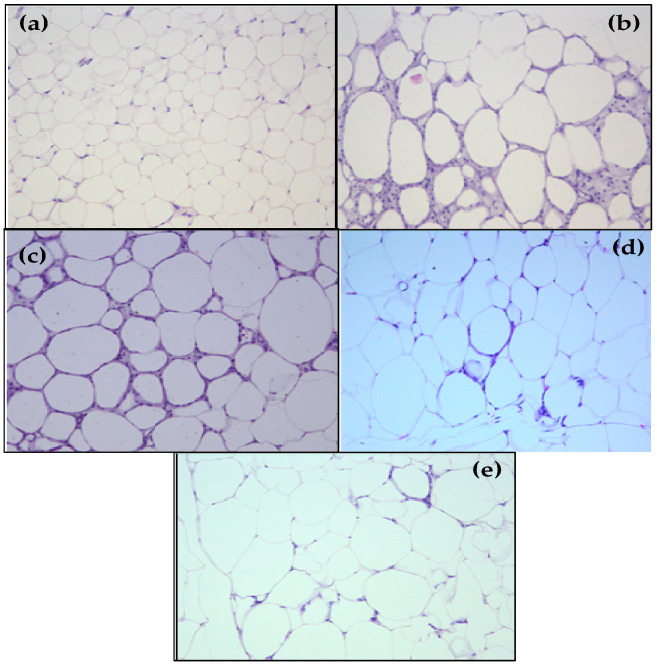

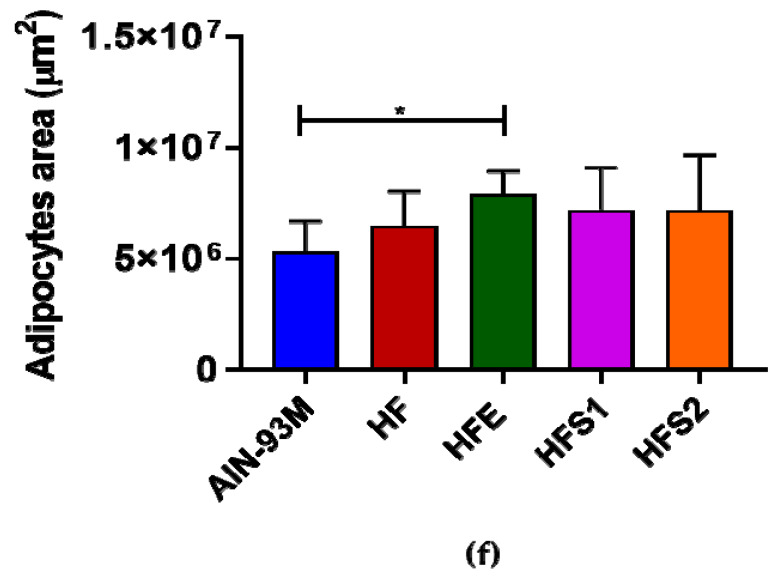
Adipocyte area of (**a**) AIN-93M diet group, (**b**) high-fat diet (HF) group, (**c**) HF supplemented with Ext at 100 mg/kg (HFE) group, (**d**) HF supplemented with substance 1 at 1 mg/kg (HFS1) group; (**e**) HF supplemented with substance 2 at 1 mg/kg (HFS2) group; and (**f**) all adipocyte areas (µm^2^). Hematoxylin and eosin staining of 5.0 µm sections of epidydimal adipose tissue. Magnification: 20×; bar scale: 100 µm. Results in (**f**) are expressed as mean ± standard error of the mean. * *p* < 0.05 compared with AIN-93M group.

The HFE group displayed an increased adipocyte area (Figure 1c) compared with the AIN-93M group (Figure 1a), and there were no differences among HF (Figure 1b), HFS1 (Figure 1d), and HFS2 (Figure 1e) groups. 

### 2.3. Ext Diminished HDL-C and Increased LDL-C Levels, Whereas S1 and S2 Diminished LDL-C and Increased HDL-C Levels Compared to HFE Group

**Figure 2:**
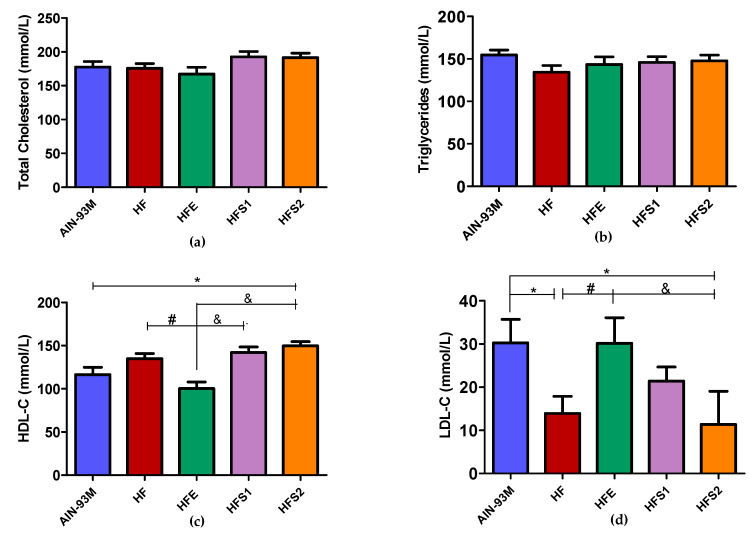
Lipid serum analysis of AIN-93M diet group, high-fat diet (HF) group, HF supplemented with Ext at 100 mg/kg (HFE) group, HF supplemented with substance 1 at 1 mg/kg (HFS1) group, and HF supplemented with substance 2 at 1 mg/kg (HFS2) group: (**a**) total cholesterol (CT; mmol/L); (**b**) triglyceride (mmol/L); (**c**) high-density lipoprotein cholesterol (HDL-C; mmol/L); (**d**) low-density lipoprotein cholesterol (LDL-C; mmol/L). Results are expressed as mean ± standard error of mean. * *p* ≤ 0.05 compared with AIN-93M, # *p* ≤ 0.05 compared with HF, and ^&^ *p* ≤ 0.05 compared with HFE. ANOVA on ranks followed by Tukey’s post-test.

At the end of the study, serum lipid analysis revealed no differences in total cholesterol (Figure 2a) and triglyceride (Figure 2b) levels among the groups. However, HFE decreased serum high-density lipoprotein cholesterol (HDL-C) levels (Figure 2c) and increased low-density lipoprotein cholesterol (LDL-C) levels (Figure 2d) compared to the HF group. The HFS2 and HFS1 group demonstrated higher HDL-C levels when compared to the HFE group (Figure 2c), while the HFS2 group displayed lower LDL-C levels compared to the HFE group (Figure 2d).

### 2.4. S1, S2, and Ext Have Not Influenced on Glycemic Metabolism 

**Figure 3:**
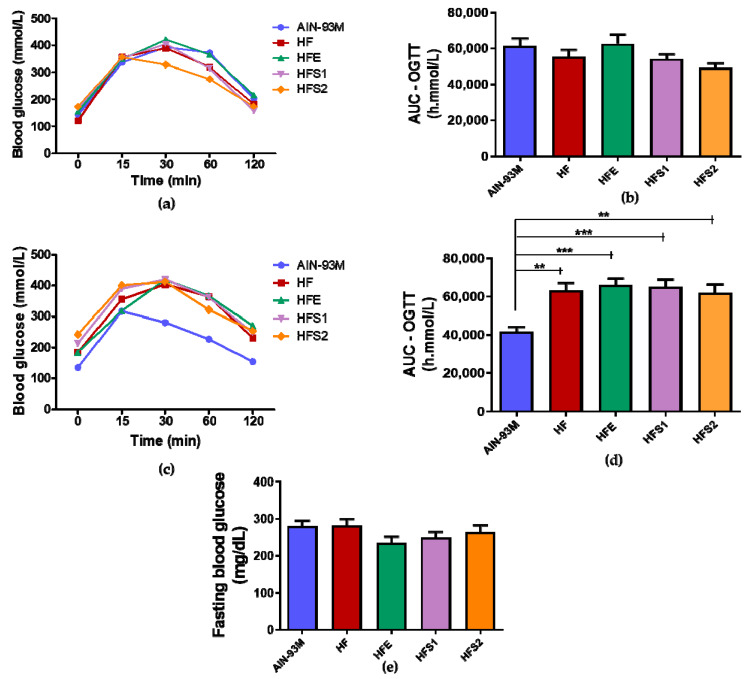
(**a**) First glycemic curve before diets and supplementation; (**b**) First oral glucose tolerance test (OGTT)–AUC graphic before diets and supplementation; (**c**) Second glycemic curve after diets and supplementation of AIN-93M diet group, high-fat diet (HF) group, HF supplemented with Ext at 100 mg/kg (HFE) group, HF supplemented with substance 1 at 1 mg/kg (HFS1) group, and HF supplemented with substance 2 at 1 mg/kg (HSF2) group; (**d**) Second OGTT–AUC graphic after diets and supplementation; (**e**) Fasting blood glucose at the end of the experiment. Results are expressed as mean ± standard error of mean. ** *p* ≤ 0.01 and *** *p* ≤ 0.001 compared with AIN-93M. ANOVA on ranks followed by Tukey’s post-test.

The first oral glucose tolerance test (OGTT) was performed before the introduction of diets and supplementation with Ext, S1, and S2; consequently, all groups responded equally without differences (Figure 3a). After 3 months of diet (HFD) and supplementation, increased area under the curve (AUC) OGTT values were observed in the HF, HFE, HFS1, and HFS2 groups compared with the AIN-93M group (Figure 3b). After euthanasia, fasting blood glucose was evaluated, and no differences were observed among the groups (Figure 3c). These findings suggest that substances S1 and S2 and Ext have no influence on glycemic parameters.

### 2.5. S1, S2 and Ext Have Not Influenced on Insulin Parameters

**Figure 4:**
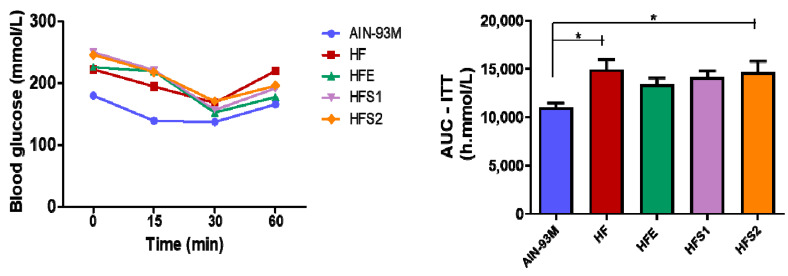
Insulin tolerance test (ITT) of AIN-93M diet group, high-fat diet (HF) group, HF supplemented with Ext at 100 mg/kg (HFE) group, HF supplemented with substance 1 at 1 mg/kg (HFS1) group, and HF supplemented with substance 2 at 1 mg/kg (HFS2) group. Results are expressed as mean ± standard error of mean. * *p* ≤ 0.05 compared with AIN-93M. ANOVA on ranks followed by Tukey’s post-test.

Insulin tolerance tests (ITTs) were performed at the end of the experiment (13th week). The HF and HFS2 groups displayed higher AUC–ITT values compared to the AIN-93M group (Figure 4). The HFS1 and HFE groups did not present differences between the AIN-93M and HF groups (Figure 4), suggesting that neither S1 and S2 nor Ext were able to influence insulin parameters after supplementation.

### 2.6. S1, S2 and Ext Have Not Attenuated Hepatic Steatosis Percentage 

**Figure 5:**
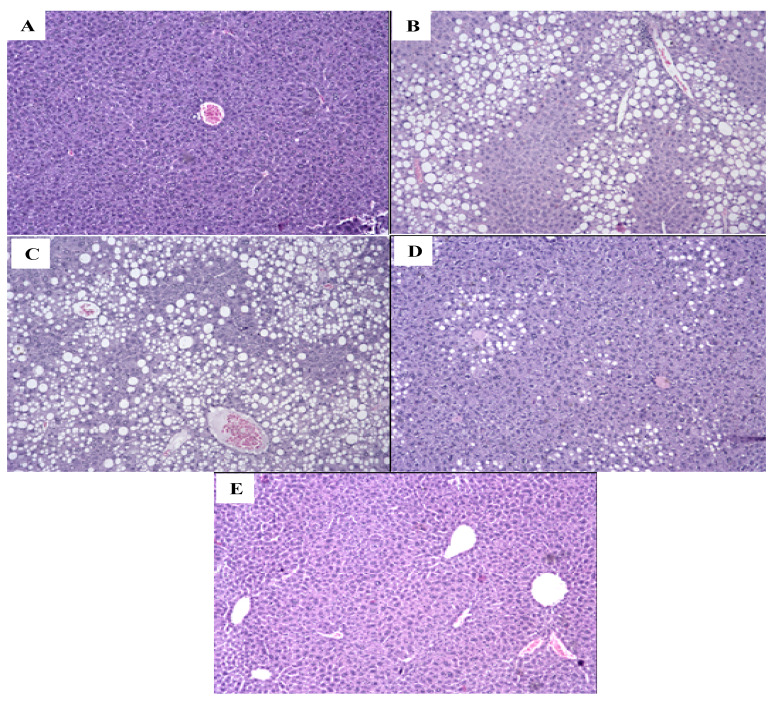
Liver histology of (**a**) AIN-93M diet group, (**b**) high-fat diet (HF) group, (**c**) HF supplemented with Ext at 100 mg/kg (HFE) group, (**d**) HF supplemented with substance 1 at 1 mg/kg (HFS1) group, and (**e**) HF supplemented with substance 2 at 1 mg/kg (HFS2) group. Hematoxylin and eosin staining of 5.0 µm sections of liver. Magnification: 20×; bar scale: 10 µm.

The AIN-93M group had a prevalence of hepatic steatosis <5% compared with the HF, HFE, and HFS1 groups (Figure 5), with no difference compared to the HFS2 group; the latter had a higher prevalence of hepatic steatosis (<5%) than the HFE group (*p* = 0.0006). When we evaluated microvesicular steatosis, our data showed that it was more prevalent in the HF, HFE, and HFS1 groups than in the AIN-93M group, while the HFE group had a higher prevalence of microvesicular steatosis than the HFS2 group (*p* = 0.003). The absence of lobular inflammation was more evident in the AIN-93M group compared to the HF and HFS1 groups (*p* = 0.003). These differences among groups were not observed for other parameters, such as apoptosis and the presence of a glycogenated nucleus. In addition, due to the absence of ballooning and Mallory’s hyaline, inferential statistical analysis was not possible (Table 3).

### 2.7. Ext, S1, and S2 Had No Influence on Pancreas Hypertrophy 

The HF group displayed differences in the islets of Langerhans, demonstrating pancreatic hypertrophy in comparison to the AIN-93M group (*p* < 0.05). The HFE, HFS1, and HFS2 groups maintained pancreatic morphology without improving hypertrophy when compared to the HF group. Inflammatory cells were not identified among the groups (*p* = 0.17) and no alterations were observed in pancreatic acini (Table 4). 

### 2.8. S1, S2, and Ext Groups Had No Influence on IL-6 and IL-10 Protein Expression in Epididymal Adipose Tissue 

**Figure 6:**
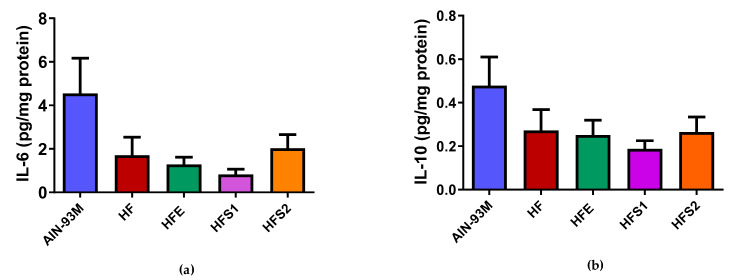
Effects of Ext, S1, and S2 on pro-inflammatory (interleukin-6 (IL-6)) and anti-inflammatory (IL-10) cytokines in epididymal adipose tissue: (**a**) IL-6 (pg/mg protein) and (**b**) IL-10 (pg/mg protein) of groups fed AIN-93M diet, high-fat diet (HF), HF supplemented with Ext at 100 mg/kg (HFE), HF supplemented with substance 1 at 1 mg/kg (HFS1), and HF supplemented with substance 2 at 1 mg/kg (HFS2). Results are expressed as mean ± standard error of mean. ANOVA on ranks followed by Tukey’s post-test.

Regarding the average values among groups, it was observed that the AIN-93M group showed a tendency to display an increased expression of IL-6 and IL-10 protein content (Figure 6), without differences when compared to the HF group. At the same time, the HFE, HFS1, and HFS2 groups showed no differences when compared to the HF and AIN-93M groups.

## 3. Discussion

Most studies that evaluate the actions of *C. xanthocarpa* in common metabolic disturbances that could be associated with obesity were not performed in diet-induced obese (DIO) animal models [15]. The present study is the first to compare the effects of *C. xanthocarpa* hydroethanolic leaf extract (Ext) and two isolated substances from the extract, 2′,4′-dihydroxy-3′,5′-dimethyl-6′-methoxychalcone (S1; chalcone) and 5-hydroxy-7-methoxy-8-methylflavanone (S2; flavanone) in HFD-fed mice to evaluate whether a multitargeted approach may be more effective as a therapeutic strategy than using isolated compounds.

Two important baseline studies observed that Ext and S1 (chalcone) were used as anti-inflammatory agents in carrageenan-induced paw edema in rodents [10] and that S2 (flavanone) displayed trypanocidal activity against *Trypanosoma cruzi* [25]. At the same time, toxic effects, such as alterations in food consumption, water intake, behavior and the weight of organs (heart, lung, spleen, liver and kidney) from Ext were not observed [10]. Based on these results that Ext did not promote signs of toxicity, we decided to compare the effects of long-term of supplementation with Ext, S1 and S2 in DIO mice. 

Other studies have demonstrated therapeutic potential using different types of extracts from diverse parts of *C. xanthocarpa* in both animal models and human clinical trials, such as antiplatelet, antithrombotic, hypoglycemic, weight loss, antihypertensive, and hypocholesterolemic activity and anti-inflammatory effects, without side effects such as gastric disturbances [10,15,16,26,27]. 

Even though Ext has presented phenols, flavonoids and tannins that were quantified following specific methodologies in the baseline study [10], our results revealed that Ext did not improve most metabolic and histological parameters and obesity features in HFD-fed mice. Despite not evaluating the mechanism of action involved in impairing metabolic responses found by Ext, it is important to emphasize that, depending on the experimental model (rodents or human clinical trials), different metabolic responses may be observed [28,29], and the different stimuli to which these models are subjected must be considered, such as different types of extracts, as observed from *C. xanthocarpa* leaf aqueous extracts [16,17,25], time and dose of treatment, diet, genetic factors, age, and gender, which may influence metabolic responses [30,31,32,33].

Chalcones and flavanones are found in a variety of plant species and belong to the flavonoid family. These compounds have gained attention due to their pharmacological activity [18]. We tested S1 (chalcone) and S2 (flavanone), which were isolated from *C. xanthocarpa* hydroethanolic leaf extract. We observed that S1 and S2 did not improve obesity features, including body weight gain and visceral adiposity. However, our main results demonstrated that S1 (chalcone) was able to diminish food intake, suggesting that this compound could be a potential substance for appetite control. 

The imbalance between energy expenditure and energy intake is proposed to be one of the causes of obesity; consequently, drugs to diminish energy intake or enhance energy expenditure or both without side effects is a field of interest, as natural dietary products are one option due to their potential for appetite control and as an alternative source for weight loss agents [34]. 

The homeostatic control of energy intake involves a complex system that involves both peripheral and central nervous system signs that are integrated and regulate satiation [35]. These mechanisms include the activation of anorexigenic mediators such as proopiomelanocortin (POMC), glucagon-like peptide-1 (GLP-1), and peptide YY (PYY) and the suppression of orexigenic mediators such as neuropeptide (NPY) and agouti-related peptide (AgRP) in the hypothalamus arcuate nucleus (ARC) and brainstem, which is the area that contributes to balancing energy and glucose, resulting in increased satiety (by stopping eating) and reduced food intake (by initiating eating). In addition, the appetite regulation activity of bioactive compounds in the gastrointestinal tract may decrease ghrelin in the stomach and increase, for instance, PYY and GLP-1, which encourage the pancreas to release insulin. Furthermore, leptin released from adipose tissue also affects the brain in regulating appetite [36,37]. 

Even though we observed decreased food consumption without differences in visceral adiposity promoted by S1, this substance presented lower average values, especially in adiposity index and total visceral fat, suggesting that a higher concentration or prolonged time of supplementation with S1 could be proposed to promote effects on adiposity. Thus, further elucidation will be necessary to clarify whether S1 can be considered an appetite control agent, especially considering homeostatic and hedonic mechanisms related to eating behavior, as observed in certain mechanisms related to peripheral hormones, such as leptin, ghrelin, and insulin, as well in the relationships between neural pathways involved in food reward [37].

Neither the two isolated substances nor the crude extract were able to act on glycemic and insulinemic metabolism. In addition, no differences were observed in total cholesterol and triglyceride levels. On the other hand, the HFE group diminished HDL-C and increased LDL-C levels in comparison to the HF group, reinforcing the idea that Ext could impair certain metabolic parameters instead of improving them. The HFS1 and HFS2 groups presented decreased LDL-C and increased HDL-C levels compared to the HFE and AIN-93M groups, demonstrating that S1 and S2 had no influence on lipid parameters. 

Even though the group that received the AIN-93M diet had higher LDL-C and lower HDL-C levels compared to the HFS1 and HFS2 groups, other parameters, including body weight, adiposity index, glucose, hepatic steatosis, and the islets of Langerhans hypertrophy, were satisfactory for the characterization of our obesity model. These controversial metabolic results related to AIN-93M [38,39,40] can be explained by the higher carbohydrate (55.07%) and lower protein (9.67%) content compared to HFD, which presented 43.29% carbohydrate and 13.65% protein content [40]. 

In terms of histological parameters, the HFE group displayed higher adipocyte area in the epidydimal adipose tissue in comparison to the AIN-93M group, demonstrating that adipocyte hypertrophy was observed in the supplementation with Ext. In prolonged positive energy balance conditions, adipocytes expand cell size to compensate for the need for increased lipid storage in white adipose tissue; therefore, several deleterious effects may be associated with this unhealthy expansion of white adipose tissue, which may lead to inflammation, altered adipokine secretion, fibrosis, hypoxia and mitochondrial disfunction [41]. After evaluating the expression of important pro-inflammatory and anti-inflammatory cytokines, IL-6 and IL-10, respectively, in epididymal adipose tissue, differences were not observed among the groups, indicating that these specific adipokines could be not altered in this condition. 

Langerhans islet hypertrophy was more pronounced in the HF group. These results correlate with another study that observed a marked increase in islet size in high-fat/sucrose diet-fed mice, suggesting the adaptive enlargement of β-cell mass in response to insulin resistance, a metabolic condition associated with hyperplasia and the hypertrophy of islets caused by the adaptive proliferation of β-cells to maintain blood glucose levels [42]. 

Our data showed that the HF group had a higher prevalence of hepatic steatosis, microvesicular hepatic steatosis, and inflammation. Ext had no influence on hepatic steatosis in the liver, and these results correlate with those of another study, where a *C. xanthocarpa* decoction was unable to prevent morphological alterations in the liver [43]. Even though some studies have shown that chalcones can prevent hepatic steatosis in the liver [20,44], our results demonstrated that S1 (chalcone) had no influence on hepatic and microvesicular steatosis; however, S2 (flavanone) showed a prevalence of hepatic steatosis <5% and lower microvesicular steatosis and inflammation compared to Ext without differences in comparison to HF.

S1 (chalcone), S2 (flavanone), and Ext showed their particularities in terms of metabolic effects in HFD-fed mice; natural products may not exert effects in general, but only in certain metabolic and histological parameters. Our results are in accordance with other studies demonstrating that some natural products may improve some metabolic and histological parameters and, at the same time, impair others [22,45]. In fact, one issue with studying natural products is that the effects from isolated substances may act differently from a synergistic perspective. For many years, research on natural products was focused on identifying, purifying, and finding specific concentrations that could be used as active agents of these substances; however, some studies have observed that a total extract might exert a better effect than an equivalent dose, suggesting that a multitargeted approach to therapy could be an alternative in some pathologies [46]. 

However, in our study, we observed that Ext could not improve most metabolic and histological parameters, suggesting that a multitargeted approach in terms of a synergistic effect did not respond adequately in our experimental model. These findings were discretely different from those for isolated substances S1 (chalcone) and S2 (flavanone), which did not have substantial effects on metabolic and histological parameters; however, S1 was able to diminish food intake. Thus, further investigations will be necessary to elucidate the local tissue-specific mechanisms in the expression of specific genes and proteins in adipose tissue and the central nervous system, in which S1 (chalcone) could be involved in appetite regulation, and to evaluate whether S1 could have an influence on prolonging adiposity, in terms of supplementation or standardizing doses to promote both effects on appetite control and decreased visceral fat.

## 4. Materials and Methods 

### 4.1. Plant Material and Preparation of Extract 

Leaves from *C. xanthocarpa* were collected in Itaporã County, Mato Grosso do Sul State, Brazil. Exsiccate (voucher specimen 4644) was deposited at the DDMS (Herbarium Code) Herbarium in Dourados County at the Universidade Federal da Grande Dourados (UFGD, Dourados, Brazil).

The extract was prepared, according to methodology used by da Silva et al. [10], by macerating the material in ethanol and water (70:30, *v*/*v*) at room temperature for 7 days. After this, the extract was filtered (110 mm), and the residue underwent two or more extractions using the same process. After 21 days, the filtrate was concentrated under reduced pressure and freeze-dried. The specific yield was calculated as 12% (*w*/*w*). The freeze-dried material was stored at −4 °C and protected from light. 

### 4.2. Isolation and Identification of Substances

The extract (49.8 g) was solubilized in water and ethanol (3:2 *v*/*v*) and fractionated with organic solvents (dichloromethane and ethyl acetate), resulting in two extracts: dichloromethane extract (DE) (9.2 g) and ethyl acetate extract (EAE) (13.1 g). EAE was fractionated by column chromatography with solvents with increasing polarities (dichloromethane, ethyl acetate, and acetone), leading to 91 fractions that were analyzed by thin layer chromatography (TLC) and clustered in 93 fractions. DE fractions 24–56 (511.4 mg) were purified by TLC in hexan EtOAc (8:2 v/v), resulting in isolate 1 (300.2 mg). EAE was fractionated by chromatography column (CC) in dichloromethane (DCM; EAE1, 1.7 g), DCM: EtOAc (1:1 *v*/*v*; EAEC2, 3.7 g), EtOAc (EEFC3, 2.9 g), and EtOH (EEFC4, 1.5 g). EAEC2 fraction was fractionated by TLC prepared in hexan and EtOAc (7:3 *v*/*v*), resulting in isolate 2 (307.2 mg). The extract indicated the presence of flavonoids, phenols and tannins according to da Silva et al. (2016) [10].

All isolated substances were identified by 1D and 2D NMR (Nuclear Magnetic Ressonance) spectroscopy. The last were identified as chalcone and flavanone after RMN data analysis; therefore, the analyses were compared in the literature. The chalcone was identified as 2′,4′-dihydroxy-3′,5′-dimethyl-6′-methoxychalcone (**1**) [10] and the flavanone was identified as 5-hydroxy-7-methoxy-8-methylflavanone (**2**) [25].

### 4.3. Ethics Statement

All animal experiments were submitted to and approved by the Ethics Committee on Animal Use, Federal University of Mato Grosso do Sul (protocol no. 858/2017).

### 4.4. Animal Care and Experimental Procedure

Adult male Swiss mice (*n* = 60, 8 weeks of age) were divided into five groups: (1) AIN-93M diet (*n* = 12); (2) high fat diet (HF; *n* = 12); (3) HF supplemented with *C. xanthocarpa* hydroethanolic leaf extract at 100 mg/kg (HFE) (*n* = 12); (4) HF supplemented with S1 (chalcone) at 1 mg/kg (HFS1; *n* = 12); (5) HF supplemented with S2 (flavanone) at 1 mg/kg (HFS2; *n* = 12) for 12 weeks. The extract and substances were administered daily by oral gavage. The doses used were followed according to a previous study that evaluated the use of Ext (100 mg/kg) and S1 (1 mg/kg) in carrageen-induced paw edema in rats [10]. An acute toxicity test was evaluated, and no toxicity signs were observed [10]. The standard diet was formulated according to the American Institute of Nutrition (AIN-93) for maintenance [47] and the high-fat diet (HFD) was based on the AIN-93M diet, adapted according to Lequinste et al. [48]. Each group had ad libitum access to water and food during the experimental period. The composition of the experimental diets is listed in Table 5. 

The mice were anesthetized using Isofluran and euthanized by inferior vena cava exsanguination after 8 h of fasting when they reached 20 weeks of age. The blood and organs were collected for further analysis. 

### 4.5. Body Weight and Diet Intake 

Mice were weighed twice per week to evaluate weight changes until the end of the study. Food intake was measured weekly. Feed efficiency index (FEI) was calculated [49,50], which refers to the amount of food consumed that can promote body weight gain, using Equation (1):

FEI = FBW − IBW/TF(1)

where FBW is the final body weight in grams, IBW is the initial body weight in grams, and TF is the total amount of food ingested in grams. Furthermore, the weight gain per caloric intake index (WGPCI) [49,50] was calculated to evaluate the animal’s capacity to convert consumed energy into body weight, following Equation (2):

WGPCI = FBW − IBW/kcal consumed(2)

### 4.6. Biochemical Analysis 

Serum glucose, serum triglyceride, total cholesterol, high-density lipoprotein cholesterol (HDL-C), and low-density lipoprotein cholesterol (LDL-C) levels were analyzed by the enzymatic colorimetric test (Labtest^®^, Lagoa Santa, Minas Gerais, Brazil). 

### 4.7. Oral Glucose Tolerance Test

An oral glucose tolerance test was performed at the beginning of supplementation with Ext, S1, and S2, and 3 days before animals were euthanized after 8 h of fasting. Fasting glucose was verified via flow rate (time 0) using a G-Tech^®^ glucometer (G-Tech Free, Infopia Co., Ltd., Anyang, Gyeonggi-do, South Korea). Thereafter, the animals received D-glucose (Vetec, Duque de Caxias, RJ, Brazil) at 2 g/kg of body weight by oral gavage, and blood glucose was monitored at 15, 30, 60, and 120 min after glucose administration. The area under the curve (AUC) was calculated for each animal and the mean was calculated for each experimental group [51].

### 4.8. Insulin Tolerance Test

An insulin tolerance test was performed 5 days before euthanasia. Glycemia was verified with the animals in a fed state (time 0). Then, insulin (Novorapid^®^, 100 UmL, Novo Nordisk, Bagsvaerd, Denmark) at a dose of 0.75 units per kg of animal weight was injected intraperitoneally. Blood glucose was monitored at 15, 30, and 60 min using a G-Tech^®^ glucometer (G-Tech Free). The AUC was calculated for each animal and the mean was calculated for each experimental group [51].

### 4.9. Quantification of Cytokine of Adipose Tissue

Epididymal adipose tissue was collected, weighed (100 mg), and stored at −80 °C. For total protein extraction and quantification, the tissue was thawed on ice and homogenized in 1 mL of Radioimmunoprecipitation Assay Buffer (RIPA) (RIPA lysis buffer, 10×, cat. no. 20-188, Merck, Darmstadt, Germany). A cocktail of protease inhibitors was added (Protease Inhibitor Cocktail Set, Calbiochem, cat. no. 539131, Merck, Darmstadt, Germany). The supernatant was collected after centrifugation at 4 °C and stored again at −80 °C until cytokine analysis, according to the manufacturer’s recommendations (MILLIPLEX MAP kit, Millipore, Billerica, MA, USA). Protein quantification was based on bicinchoninic acid assay (BCA) following the manufacturer’s recommendations (BCA Protein Assay kit, Merck, Darmstadt, Germany). The concentrations of IL-6 and IL-10 cytokines (MCYTOMAG-70k) were analyzed and expressed as the ratio of cytokine picograms in relation to protein content (mg of protein) [52].

### 4.10. Assessment of Body Fat and Liver Weight 

After euthanasia, the liver and fat pads of white adipose tissue (omental, epididymal, perirenal, retroperitoneal, and mesenteric) were dissected and weighed. The adiposity index was calculated by the ratio of the sum of visceral white adipose tissue (g) and the final body weight of the animal × 100 and was expressed as percentage of adiposity [53].

### 4.11. Histological Analysis: Epididimal Adipose Tissue, Liver, and Pancreas

Samples of epididymal adipose tissue, liver, and pancreas were fixed with 10% formalin solution. After fixation, the specimens were dehydrated, embedded in paraffin, cut in a microtome to a thickness of 5 mm each, and stained with hematoxylin and eosin [54,55]. An expert pathologist performed the histological analysis of the liver and classified the samples according to a score system by Kleiner et al. [56]. A histological analysis of the pancreas followed the architecture of pancreas evaluation, according to changes in the islets of Langerhans [57,58]. Images of the adipocyte area of the epididymal adipose tissue were taken using a Leica DFC 495 digital camera system (Leica Microsystems, Wetzlar, Germany) integrated into a Leica DM 5500B microscope (Leica Microsystems, Wetzlar, Germany) with a magnification of 20×. The images were analyzed using the Leica Application Suite software, version 4.0 (Leica Microsystems, Wetzlar, Germany), and the mean area of 100 adipocytes per sample was determined [14].

### 4.12. Statistical Analysis 

The results were expressed as mean ± mean standard error (MSE). Statistically significant differences in mean values among the groups were assessed using ANOVA followed by Tukey’s post-test on parametric results, with a Kruskal–Wallis test followed by Dunn’s post-test on nonparametric results. A chi-square test was used to evaluate the histological analysis, followed by Bonferroni’s post-test. A level of *p* ≤ 0.05 was considered statistically significant. Statistical analysis was performed using Jandel Sigma Stat, version 3.5 (Systat Software Inc., San Jose, CA, USA), and Sigma Plot, version 12.5 (Systat Software Inc.).

## 5. Conclusions

Most of the studies that evaluated the effects of extracts from *C. xanthocarpa* observed that a multitargeted approach could be useful in therapy for metabolic dysfunctions associated (or not) with obesity. In contrast to our findings, Ext did not improve metabolic, histologic, or obesity parameters. We observed that substances S1 (chalcone) and S2 (flavanone), isolated from the extract, did not show substantial effects on obesity features and metabolic parameters. In fact, our main findings revealed that S1 (chalcone) was able to decrease food intake, demonstrating that this substance could be a potential appetite regulatory agent. Thus, further investigations will be necessary to evaluate the principal genes and protein expressions in adipose tissue and the central nervous system involved in the regulation of appetite control and evaluate whether S1 could have an influence on prolonging adiposity, in terms of supplementation or standardizing doses to promote both effects on appetite control and decreased visceral fat.

## Figures and Tables

**Table 1:** Initial and final body weight, body weight gain, total visceral fat, and adiposity index of groups fed American Institute of Nutrition (AIN-93M) diet, high-fat diet (HF), HF supplemented with extract (Ext) at 100 mg/kg (HFE), HF supplemented with substance 1 (HFS1) at 1 mg/kg, and HF supplemented with substance 2 (HFS2) at 1 mg/kg from the first to 13th week.

Parameter			Groups		
	AIN-93M	HF	HFE (100 mg/kg)	HFS1 (1 mg/kg)	HFS2 (1 mg/kg)
Initial body weight (g)	36.08 ± 1.11	37.45 ± 1.13	38.09 ± 1.17	35.83 ± 0.89	36.16 ± 1.33
Final body weight (g) #	37.33 ± 1.18	45.09 ± 1.80	49.09 ± 1.86 ***	42.41 ± 1.78	43.75 ± 2.81
Body weight gain (g) #	1.25 ± 0.55	7.63 ± 0.83 **	11.00 ± 1.10 ***	6.58 ± 1.20	7.58 ± 1.67
Total visceral fat (g)	2.326 ± 0.239	4.000 ± 0.351 *	4.796 ± 0.229 ***	3.708 ± 0.419	4.186 ± 0.534 **
Adiposity index (%)	6.091 ± 0.537	8.721 ± 0.560 *	9.440 ± 0.292 **	8.504 ± 0.739	9.169 ± 0.851 *

Values represent mean ± standard error of the mean. In the same line, * *p* ≤ 0.05, ** *p* ≤ 0.01, and *** *p* ≤ 0.001 compared with AIN-93M group. ANOVA on ranks followed by Tukey’s post-test, # median Dunn’s post-test.

**Table 2:** Food intake at fourth, eighth, and 13th weeks: total food intake, daily caloric intake, feed efficiency index (FEI), and weight gain per caloric intake coefficient (WGPCI) of groups fed AIN-93M diet, high-fat diet (HF), HF supplemented with Ext at 100 mg/kg (HFE), HF supplemented with substance 1 (HFS1) at 1 mg/kg, and HF supplemented with substance 2 (HFS2) at 1 mg/kg.

Parameters			Groups		
	AIN-93M	HF	HFE (100 mg/kg)	HFS1 (1 mg/kg)	HFS2 (1 mg/kg)
Food intake (4th week) (g)	112.33 ± 2.59	95.90 ± 2.64 *	98.09 ± 3.55	93.41 ± 3.24 **	96.16 ± 5.58 *
Food intake (8th week) (g) #	102.91 ± 3.47	86.60 ± 2.02	85.63 ± 1.75 *	84.16 ± 1.44 *	82.91 ± 4.03 *
Food intake (13th week) (g) #	135.50 ± 5.46	107.16 ± 4.67 *	107.72 ± 2.83	103.75 ± 2.01 *^,&^	100.75 ± 5.41 *
Total food intake (total g) #	350.75 ± 10.89	292.54 ± 10.11 *	291.45 ± 7.65	281.33 ± 5.84 *	279.83 ± 14.66 *
Daily caloric intake (kcal/day)	14.80 ± 0.46	17.22 ± 0.59 *	17.16 ± 0.45 *	16.57 ± 0.34	16.47 ± 0.85
FEI	37.22 ± 1.18	44.96 ± 1.79	48.96 ± 1.86 ***	42.29 ± 1.78	43.62 ± 2.81
WGPCI	36.72 ± 1.29	45.06 ± 1.80 *	49.06 ± 1.86 ***	42.39 ± 1.78	43.72 ± 2.81

Values represent mean ± standard error of the mean. In the same line, * *p* ≤ 0.05, ** p ≤ 0.01, and *** *p* ≤ 0.001 compared with AIN-93M group; ^&^ *p* ≤ 0.05 compared with HF. ANOVA on ranks followed by Tukey’s post-test, # median Dunn’s post-test.2.3. S1 and S2 did not influence adipocyte size; Ext increased adipocyte area

**Table 3:** Liver histopathological analysis following scores on hepatic steatosis, microvesicular steatosis, lobular inflammation, ballooning, Mallory’s hyaline, apoptosis, glycogenated nucleus of AIN-93M diet group, high-fat diet (HF) group, HF supplemented with Ext at 100 mg/kg (HFE) group, HF supplemented with substance 1 at 1 mg/kg (HFS1) group, and HF supplemented with substance 2 at 1 mg/kg (HFS2) group.

Parameters			Groups		
	AIN-93M (*n* = 12)	HF (*n* = 11)	HFE (100 mg/kg) (*n* = 11)	HFS1 (1 mg/kg) (*n* = 10)	HFS2 (1 mg/kg) (*n* = 12)
Hepatic steatosis (*p* = 0.0006 *)					
<5%	100.0 (12)	18.2 (2)	0.0 (0)	30.0 (3)	58.3 (7)
5 to 33%	0.0 (0)	27.2 (3)	36.4 (4)	30.0 (3)	16.7 (2)
34 to 66%	0.0 (0)	36.4 (4)	27.3 (3)	40.0 (4)	8.3 (1)
>66%	0.0 (0)	18.2 (2)	36.4 (4)	0.0 (0)	16.7 (2)
**Microvesicular steatosis (*p* = 0.003)**					
Absence	100.0 (12)	81.8 (9)	36.4 (4)	60,0 (6)	91.7 (11)
Presence	0.0 (0)	18.2 (2)	63.6 (7)	40.0 (4)	8.3 (1)
**Lobular inflammation (*p* = 0.003)**					
Absence	100.0 (12)	63.6 (7)	9.1 (1)	30.0 (3)	50.0 (6)
<1 focus/field	0.0 (0)	36.4 (4)	72.7 (8)	50.0 (5)	33.3 (4)
2–4 foci/field	0.0 (0)	0.0 (0)	18.2 (2)	20.0 (2)	16.7 (2)
**Ballooning ^a^**					
Absence	100.0 (12)	100.0 (11)	100.0 (11)	100.0 (10)	100.0 (12)
**Mallory’s hyaline ^a^**					
Absence	100.0 (12)	100.0 (11)	100.0 (11)	100.0 (10)	100.0 (12)
**Apoptosis (*p* = 0.48)**					
Absence	100.0 (12)	100.0 (11)	90.9 (10)	90.0 (9)	100.0 (12)
Presence	0.0 (0)	0.0 (0)	9.1 (1)	10.0 (1)	0.0 (0)
**Glycogenated nucleus**					
None/rare	100.0 (12)	100.0 (11)	81.8 (9)	100.0 (10)	100.0 (12)
Some	0.0 (0)	0.0 (0)	18.2 (1)	0.0 (0)	0.0 (0)

^a^ Inferential statistical analysis not available due to the absence of values in the analyzed categories. * Data presented as relative frequency (absolute frequency). Value of p on chi-square test.

**Table 4:** Analysis of pancreatic alterations observed in groups fed AIN-93M diet, high-fat diet (HF), HF supplemented with Ext at 100 mg/kg (HFE), HF supplemented with substance 1 at 1 mg/kg (HFS1), and HF supplemented with substance 2 at 1 mg/kg (HFS2).

Parameters			Experimental Groups % (*n*)		
	AIN-93M (*n* = 12)	HF (*n* = 11)	HFE (100 mg/kg) (*n* = 11)	HFS1 (1 mg/kg) (*n* = 10)	HFS2 (1 mg/kg) (*n* = 11)
Islets of Langerhans (*p* = 0.04 *)					
No alteration	100.0 (12)	45.5 (11) *	63.6 (7)	80.0 (8)	81.8 (9)
Hypertrophy	0.0 (0)	54.6 (6)	36.4 (4)	20.0 (2)	18.2 (2)
Pancreatic acini (**)					
No alteration	100.0 (12)	100.0 (11)	100.0 (11)	100.0 (10)	100.0 (11)
**Inflammatory cells (*p* = 0.17)**					
No alteration Peri-insulitis	100.0 (12)0.0 (0)	81.8 (9)18.2 (2)	81.8 (9)18.2 (2)	100.0 (10)0.0 (0)	100.0 (11)0.0 (0)

* Significant association by chi-square test followed by Bonferroni’s post-test. ** Inferential statistical analysis not feasible due to the absence of values in the categories analyzed.

**Table 5:** Composition of experimental diets (g/kg of diet).

Experimental Groups (g/kg)	AIN-93M Diet	High-Fat Diet (HF)
Composition (g/kg)		
Lard	–	320.00
Corn starch	630.692	320.692
Casein (>85% of protein)	140.00	140.00
Cellulose	50.00	50.00
Vitamin mix	10.00	10.00
Mineral mix	35.00	35.00
Soybean oil	40.00	20.00
L-cistin	1.80	1.80
Choline bitartrate	2.50	2.50
Sucrose	100.00	100.00
Tert-butylhydroquinone	0.008	0.008
Energy (kcal/g)	3802.8	5302.8
Carbohydrates (%)	75.81	31.73
Protein (%)	14.73	10.56
Lipids (%)	9.47	57.71
Calories/g of diet	3.80	5.30
